# Right Ventricular Outflow Tract Obstruction Diagnosed after Percutaneous Ventricular Septal Defect Device Closure

**Published:** 2015-07-03

**Authors:** Ali Hosseinsabet

**Affiliations:** Tehran Heart Center, Tehran University of Medical Sciences, Tehran, Iran.

**Keywords:** *Ventricular outflow obstruction*, *Septal occlude device*, *Diagnosis*

A 23-year-old woman referred to our echocardiography laboratory for follow-up transthoracic echocardiography (TTE). She had undergone perimembranous ventricular septal defect (VSD, size = 6 mm) closure with the Amplatzer device one month previously. Right catheterization before VSD closure revealed no right ventricular outflow tract (RVOT) stenosis. At the time of echocardiography, she was asymptomatic. TTE showed increased wall thickness and a narrowed RVOT (10 mm at diastole and 6 mm at systole) with a turbulent flow at device replacement (Figure 1). Although maximal alignment with this turbulent flow was not possible, a peak pressure gradient of 36 mmHg was obtained via continuous wave. It is feasible that there was some degree of RVOT obstruction before VSD device closure, which was intensified after device placement. The hypertrophied RVOT walls or the hypertrophied muscle bundle resulted in a narrow RVOT; and with VSD device placement at this narrow tract, the stenosis became more prominent. Accordingly, when VSD closure by device is planned, the possibility of stenosis in a narrowed RVOT should be taken into account. Also, a comprehensive cardiac evaluation in all patients with congenital heart disease should be considered to find other associated anomalies.

**Figure 1:**
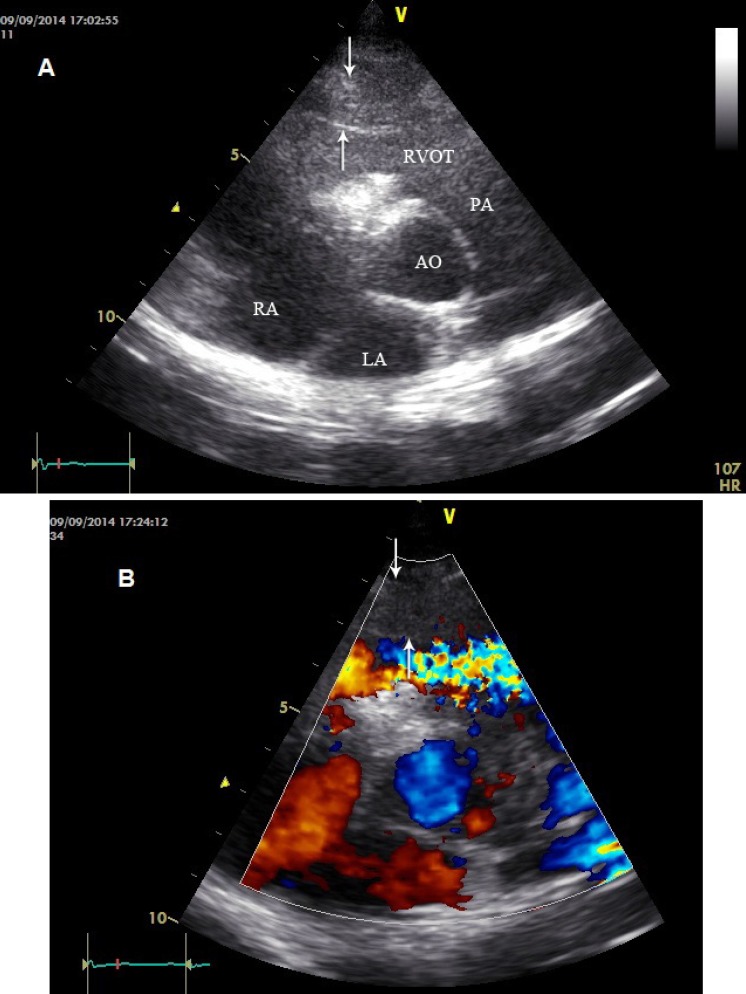
A) Hypertrophy (between two arrows) of the right ventricular outflow tract (RVOT) anterior wall in the parasternal short-axis view at the level of the aortic valve and the ventricular septal defect (VSD) device occluder in two-dimensional echocardiography. B) Turbulent flow at the RVOT at the VSD device site in color-flow Doppler in the parasternal short-axis view at the level of the aortic valve

RA, Right atrium; LA, Left atrium; AO, Aorta; PA, Pulmonary artery

***To watch the following videos, please refer to the relevant URLs:***

Video 1. Hypertrophy of the anterior wall of the right ventricular outflow tract in the parasternal short-axis view at the level of the aortic valve and the ventricular septal defect device occluder in two-dimensional echocardiography

http://jthc.tums.ac.ir/index.php/jthc/article/view/779/503

Video 2.Turbulent flow at the right ventricular outflow tract at the ventricular septal defect device site in color-flow Doppler

http://jthc.tums.ac.ir/index.php/jthc/article/view/779/502

## Notes:

***This paper should be cited as: ***Hosseinsabet A. Right Ventricular Outflow Tract Obstruction Diagnosed after Percutaneous Ventricular Septal Defect Device Closure. J The Univ Heart Ctr 2015;10(3):165-166.

